# Genetic diversity of clinical *Pseudomonas aeruginosa* isolates in a public hospital in Spain

**DOI:** 10.1186/1471-2180-13-138

**Published:** 2013-06-18

**Authors:** Margarita Gomila, Maria del Carmen Gallegos, Victoria Fernández-Baca, Antonio Pareja, Margalida Pascual, Paz Díaz-Antolín, Elena García-Valdés, Jorge Lalucat

**Affiliations:** 1 Microbiología, Hospital Son Llàtzer, Palma de Mallorca, Illes Balears 07198, Spain; 2 Unidad de Epidemiología y control de infecciones, Hospital Son Llàtzer, Palma de Mallorca, Illes Balears 07198, Spain; 3 Microbiología, Departamento de Biología, Universidad de las Islas Baleares, and Instituto Mediterráneo de Estudios Avanzados (CSIC-UIB), Palma de Mallorca, Illes Balears 07122, Spain

**Keywords:** Pseudomonas aeruginosa, Multilocus sequence typing, Multiresistant, Clinical isolates, Population structure

## Abstract

**Background:**

*Pseudomonas aeruginosa* is an important nosocomial pathogen that exhibits multiple resistances to antibiotics with increasing frequency, making patient treatment more difficult. The aim of the study is to ascertain the population structure of this clinical pathogen in the Hospital Son Llàtzer, Spain.

**Results:**

A significant set (56) of randomly selected clinical *P*. *aeruginosa* isolates, including multidrug and non-multidrug resistant isolates, were assigned to sequence types (STs) and compared them with their antibiotic susceptibility profile classified as follows: extensively drug resistant (XDR), multidrug resistant (MDR) and non-multidrug resistant (non-MDR). The genetic diversity was assessed by applying the multilocus sequence typing (MLST) scheme developed by Curran and collaborators, and by the phylogenetic analysis of a concatenated tree. The analysis of seven loci, *acsA*, *aroE*, *guaA*, *mutL*, *nuoD*, *ppsA* and *trpE*, demonstrated that the prevalent STs were ST-175, ST-235 and ST-253. The majority of the XDR and MDR isolates were included in ST-175 and ST-235. ST-253 is the third in frequency and included non-MDR isolates. The 26 singleton sequence types corresponded mainly to non-MDR isolates. Twenty-two isolates corresponded to new sequence types (not previously defined) of which 12 isolates were non-MDR and 10 isolates were MDR or XDR.

**Conclusions:**

The population structure of clinical *P*. *aeruginosa* present in our hospital indicates the coexistence of nonresistant and resistant isolates with the same sequence type. The multiresistant isolates studied are grouped in the prevalent sequence types found in other Spanish hospitals and at the international level, and the susceptible isolates correspond mainly to singleton sequence types.

## Background

*Pseudomonas aeruginosa* is a non-fermenting Gram-negative bacterium that is widely distributed in nature. The minimum nutritional requirements, tolerance to a wide variety of physical conditions and intrinsic resistance against many antibiotics explain its role as an important nosocomial pathogen. Certain bacterial clones have been distributed worldwide and, in most cases, associated with multiresistance patterns [1-3]. Because the number of active antibiotics against *P*. *aeruginosa* is limited, it is a priority to perform a strict and regular follow up of the resistance patterns in individual hospitals.

In the microbiology laboratory of the Hospital Son Llàtzer (Mallorca, Spain) the number of isolates of *P*. *aeruginosa* is increasing annually. In 2010, the number of isolates of *P*. *aeruginosa* was 1174, being the second pathogen isolated after *Escherichia coli*. When the *P*. *aeruginosa* resistance pattern of the *P*. *aeruginosa* isolates from this hospital were compared with the latest Spanish surveillance study of antimicrobial resistance [4], it was revealed that the resistance levels of the isolates in our hospital were higher against all of the antibiotics commonly used in the treatment of infections caused by *P*. *aeruginosa*, contributing to therapeutic difficulties.

The introduction of molecular techniques has led to significant progress in both bacterial identification and typing. In *P*. *aeruginosa*, several schemes for molecular typing have been used, such as ribotyping [5], PCR-based fingerprinting [6], or pulsed-field gel electrophoresis (PFGE) [7], which is considered the ‘gold standard’ technique. Curran et al. (2004) developed a multilocus sequence typing (MLST) scheme that discriminates *P*. *aeruginosa* isolates by differences in the sequences of seven genes: *acsA*, *aroE*, *guaA*, *mutL*, *nuoD*, *ppsA* and *trpE*, providing a good comprehensive database that allows the comparison of results obtained in different locations for different sample types [8]. Since this work, MLST has been applied in several studies of *P*. *aeruginosa* to better understand the epidemiology of infections in patients with cystic fibrosis and to study multiresistant clones.

The main objective of our study is to characterise the isolates of *P*. *aeruginosa* analysed routinely in the Hospital Son Llàtzer at the molecular level. A significant set of randomly selected clinical isolates (fifty-six), including multidrug and non-multidrug resistant isolates, was further studied to determine the population structure of this clinical pathogen in our hospital and to compare it with other Spanish and international multicentre surveillance studies.

## Methods

### *P*. *aeruginosa* culture collection

A total of 56 isolates of *P*. *aeruginosa* from 53 specimens recovered from 42 patients of the Hospital Son Llàtzer were randomly selected between January and February 2010. Three samples showed two distinct colony morphologies, and both types of each isolate were studied by MLST to establish possible differences between them (these morphologies are labelled by the number of the isolate, followed by the letters a or b). Isolates from different origins were taken as part of standard care (Table 1). The hospital is a tertiary teaching hospital with 377 beds and serves a catchment population of approximately 250,000 inhabitants. All of the *P*. *aeruginosa* isolates were isolated and cultured on Columbia agar with 5% sheep blood (bioMérieux, Marcy d’Etoile, France). The cultivation and incubation times of the plates were performed under routine laboratory conditions (24 h at 37°C). The study was approved by the research board of our hospital. Individual patient’s consent was not sought as isolates were derived from routine diagnostics and as data were processed anonymously.

**Table 1:** List of the alleles and sequence type, origin of the sample, antibiotic pattern and number of patient for each isolate

**Isolates**	**Patient**	**Sample origin**^**a**^	**STs**	***acs*A**	***aro*E**	***gua*A**	***mut*L**	***nuo*D**	***pps*A**	***trp*E**	**Antibiotic pattern**^**b**^
PaC1	1	W	ST-1138*	5	3	79	5	1	7	47	XDR
PaC49	1	RS	ST-1191*	45	148*	15	35	53	106*	3	MDR
PaC51	1	RS	ST-1147*	17	3	5	3	1	6	26	non-MDR
PaC52	1	W	ST-1138*	5	3	79	5	1	7	47	XDR
PaC2	2	W	ST-175	28	22	5	3	3	14	19	XDR
PaC3	3	R	ST-235	38	11	3	13	1	2	4	non-MDR
PaC4	4	R	ST-800	17	22	11	3	3	15	3	non-MDR
PaC5	5	W	ST-108	39	5	20	5	1	6	31	XDR
PaC7	6	R	New-1	28	5	36	-	3	13	7	MDR
PaC16	6	R	New-2	28	5	20	-	3	13	7	MDR
PaC9	7	U	ST-274	23	5	11	7	1	12	7	XDR
PaC10	8	U	ST-1139*	28	22	20	3	3	14	19	XDR
PaC19	8	U	ST-175	28	22	5	3	3	14	19	XDR
PaC32	8	RS	ST-175	28	22	5	3	3	14	19	XDR
PaC40	8	R	ST-175	28	22	5	3	3	14	19	XDR
PaC11	9	U	ST-235	38	11	3	13	1	2	4	XDR
PaC24	9	U	ST-235	38	11	3	13	1	2	4	XDR
PaC12	10	R	ST-1140*	38	11	20	13	1	2	4	XDR
PaC14	11	R	ST-1141*	113	4	5	67	1	17	26	non-MDR
PaC15	12	R	ST-1142*	35	11	112	16	1	6	42	MDR
PaC17	13	R	ST-1186*	39	5	20	59*	1	20	15	non-MDR
PaC18a	14	U	ST-1143*	8	5	9	3	1	4	9	non-MDR
PaC18b	14	U	ST-1143*	8	5	9	3	1	4	9	non-MDR
PaC20	15	U	ST-1144*	39	10	1	3	4	6	7	non-MDR
PaC21	16	R	ST-175	28	22	5	3	3	14	19	XDR
PaC23	17	U	ST-1187*	35	22	5	3	1	104*	4	non-MDR
PaC25a	18	U	ST-207	47	4	5	33	1	6	40	non-MDR
PaC25b	18	U	ST-207	47	4	5	33	1	6	40	non-MDR
PaC26	19	R	ST-235	38	11	3	13	1	2	4	XDR
PaC27	20	R	ST-274	23	5	11	7	1	12	7	non-MDR
PaC28	21	U	ST-1188*	69*	147*	5	11	2	15	2	MDR
PaC29	22	R	ST-1145*	15	5	1	1	1	12	1	non-MDR
PaC30	23	PS	ST-244	17	5	12	3	14	4	7	non-MDR
PaC31	24	U	ST-499	11	5	7	27	2	7	33	MDR
PaC33	25	R	ST-175	28	22	5	3	3	14	19	XDR
PaC34	26	R	ST-179	36	27	28	3	4	13	7	MDR
PaC35	27	U	ST-235	38	11	3	13	1	2	4	MDR
PaC36a	28	R	ST-175	28	22	5	3	3	14	19	XDR
PaC36b	28	R	ST-175	28	22	5	3	3	14	19	MDR
PaC37	29	B	ST-175	28	22	5	3	3	14	19	XDR
PaC38	29	B	ST-175	28	22	5	3	3	14	19	XDR
PaC39	30	U	ST-508	15	5	11	3	2	4	3	non-MDR
PaC41	31	U	ST-1189*	72*	5	11	72	3	105*	3	non-MDR
PaC42	32	W	ST-253	4	4	16	12	1	6	3	MDR
PaC56	32	B	ST-253	4	4	16	12	1	6	3	MDR
PaC43	33	R	ST-235	38	11	3	13	1	2	4	XDR
PaC44	34	U	ST-316	13	8	9	3	1	6	9	non-MDR
PaC45	35	U	ST-175	28	22	5	3	3	14	19	XDR
PaC46	36	R	ST-1190*	45	148*	15	35	53	106*	19	MDR
PaC47	37	R	ST-175	28	22	5	3	3	14	19	XDR
PaC48	38	R	ST-253	4	4	16	12	1	6	3	non-MDR
PaC54	38	R	ST-253	4	4	16	12	1	6	3	non-MDR
PaC50	39	US	ST-1146*	5	11	57	33	1	6	3	non-MDR
PaC53	40	U	ST-1148*	16	5	12	3	2	1	18	non-MDR
PaC55	41	R	ST-179	36	27	28	3	4	13	7	non-MDR
PaC57	42	R	ST-1149*	18	11	57	5	1	20	26	non-MDR

Asterisk mark (*) indicates the new alleles, new alleles combinations, and new sequence types described in this study. -, gene not amplified/sequenced.

^a^Sample origin: blood (B), wound (W), urine (U), respiratory (R), rectal smear (RS), urethral smear (US), prepuce smear (PS).

^b^The isolates were classified according to the resistance pattern, as multidrug-resistant (MDR, non-susceptible to ≥1 agent in ≥3 antibiotic categories), extensively drug-resistant (XDR, non-susceptible to ≥ 1 agent in all but ≤2 antibiotic categories) and non-multidrug resistant (non-MDR).

### Phenotypic and antibiotic susceptibility characterisations

The 56 isolates were biochemically and phenotypically characterised using the authomatized VITEK®2 GN method (bioMériux, Marcy d’Etoile, France) and the oxidase reaction test. Their antibiogram profiles were established by the disk diffusion method on Mueller-Hinton agar plates (bioMérieux, Marcy d’Etoile, France) following CLSI recommendations for all antibiotics, except for fosfomycin which followed the French Microbiology Society recommendations [9,10]. Borderline values were assessed by the E-test method (bioMérieux, Marcy d’Etoile, France). The antibiotics tested were amikacin, aztreonam, cefepime, ceftazidime, ciprofloxacin, colistin, gentamicin, fosfomycin, imipenem, levofloxacin, meropenem, piperacillin-tazobactam and tobramycin. For the isolates resistant to imipenem and/or meropenem, the determination of metallo-β-lactamases (MBLs) using E-test strips with Imipenem-EDTA was performed (bioMérieux, Marcy d’Etoile, France). The classification of multiresistance was performed according to Magiorakos et al. [11]. The isolates were classified according to the resistance pattern as multidrug resistant (MDR, non-susceptible to at least one agent in three or more antimicrobial categories), extensively drug resistant (XDR, non-susceptible to at least one agent in all but two or fewer antimicrobial categories; i.e. bacterial isolates remain susceptible to only one or two categories), pandrug-resistant (PDR, non-susceptible to all agents in all antimicrobial categories), and non-multidrug resistant (non-MDR).

### DNA extraction: PCR amplification and DNA sequencing

Bacterial genomic DNA for PCR amplification was obtained as previously described [12]. The housekeeping genes *acsA*, *aroE*, *guaA*, *mutL*, *nuoD*, *ppsA* and *trpE* were amplified and sequenced for the 56 isolates using the primers described previously [8]. The PCR conditions have been slightly modified. The reactions were performed using an Eppendorf thermocycler, with an initial denaturation step at 96°C 2 min, followed by 35 cycles of denaturation at 96°C for 1 min for all of the genes, a primer annealing temperature, depending on the gene (55–58°C for *aroE* and *nuoD*; 58°C for *acs*A and *gua*A; and 58–60°C for *mut*L, *ppsA* and *trp*E), for 1 min and a primer extension at 72°C for 1 min for all of the genes, with the exception of *aro*E (1.5 min). A final elongation step was performed at 72°C for 10 min. The PCR amplification reactions were performed as previously described [12]. The amplified products were purified with Multiscreen HTS PCR 96-well filter plates (Millipore). Sequence reactions were carried out using the ABI Prism BigDye Terminator version 3.1 and the sequences were read with an automatic sequence analyser (3130 genetic analyzer; Applied Biosystems).

### Sequence analysis and allele and nucleotide diversity

Sequence analysis was performed as described previously [12]. Individual phylogenetic trees and concatenated analyses of the sequenced gene fragments were constructed [12]. The allelic and nucleotide diversities were calculated from the gene sequences using the DnaSP package, version 3.51 [13]. For each isolate, the combination of alleles obtained at each locus defined its allelic profile or sequence type (ST). The ST and allele assignment were performed at the *P*. *aeruginosa* MLST website (http://pubmlst.org/paeruginosa/). If a sequence did not match with an existing locus in the database, it was designated as a “new” allele. Moreover, the new STs that did not match any allele combination in the database were also numbered as “new”. The clustering of the STs and the split decomposition were performed as previously described [12]. The new nucleotide sequences of each different allele of each locus determined in this study and the new sequence types were sent to curator Eleanor Pinnock for introduction into the *P*. *aeruginosa* MLST website (http://pubmlst.org/paeruginosa/).

The diversity and rarefaction indexes for the statistical analysis were calculated using the PAST v.2.0 program [14]. The coverage index (C) was calculated as C = 1 - (n/N), with n being the number of sequence types, and N the number of strains analysed.

## Results

### Description of the bacterial isolates

In total, 227 *P*. *aeruginosa* isolates were obtained from 145 patients between January and February 2010. The antibiotic resistance patterns for the isolates were 21.4% XRD, 17.2% MRD and 61.4% non-MRD. In total, 56 *P*. *aeruginosa* isolates from 53 specimens were randomly chosen from the different groups of antibiotic resistance and further studied. Three of them showed two different colony morphologies, and both types were studied by MLST. The isolates were classified according to the resistance pattern as 21.4% MDR, 37.5% XDR and 41.1% non-MDR. The antibiotic pattern and the individual profiles are shown in Table 2.

**Table 2:** Susceptibility antibiotic pattern for each *Pseudomonas aeruginosa* isolate analysed

		**ANTIBIOTICS**^**b**^													
**Strain**	**Antibiotic pattern**^**a**^	**AMK**	**GEN**	**TOB**	**IMP**	**MEM**	**CAZ**	**FEP**	**CIP**	**LVX**	**ATM**	**TZP**	**COL**	**FOS**	**IMP-EDTA**
PaC1	XDR	S	S	S	R	R	R	R	R	R	R	R	S	R	Neg
PaC49	MDR	S	S	S	R	R	I	R	S	S	R	S	S	S	Neg
PaC51	non-MDR	S	S	S	R	R	S	S	S	S	S	S	S	R	Neg
PaC52	XDR	S	I	S	R	R	R	R	R	R	R	R	S	R	Neg
PaC2	XDR	S	R	R	R	R	R	R	R	R	I	R	S	R	Neg
PaC3	non-MDR	S	S	S	S	S	S	S	R	R	S	S	S	R	-
PaC4	non-MDR	S	I	S	S	S	S	S	S	S	S	S	S	S	-
PaC5	XDR	S	S	S	R	R	R	R	S	I	R	R	S	R	Neg
PaC7	MDR	S	S	S	R	R	R	R	R	R	R	R	S	S	Neg
PaC16	MDR	S	S	S	R	I	R	R	R	R	R	R	S	S	Neg
PaC9	XDR	S	I	S	R	R	S	R	R	R	R	S	S	R	Neg
PaC10	XDR	S	R	R	R	R	R	R	R	R	I	R	S	R	Neg
PaC19	XDR	S	R	R	R	R	R	R	R	R	S	R	S	R	Neg
PaC32	XDR	S	R	R	R	R	R	R	R	R	I	R	S	R	Neg
PaC40	XDR	S	R	R	R	R	R	R	R	R	S	R	S	R	Neg
PaC11	XDR	R	R	R	S	S	R	R	R	R	R	R	S	R	Neg
PaC24	XDR	R	R	R	S	S	R	R	R	R	R	R	S	R	Neg
PaC12	XDR	S	R	R	R	R	R	R	R	R	R	R	S	R	Pos
PaC14	non-MDR	S	I	S	S	S	S	S	S	S	S	S	S	R	-
PaC15	MDR	S	I	S	S	S	S	S	S	S	I	S	S	R	-
PaC17	non-MDR	S	S	S	S	S	S	S	S	S	S	S	S	R	-
PaC18a	non-MDR	S	I	S	S	S	S	S	S	S	S	S	S	R	-
PaC18b	non-MDR	S	S	S	S	S	S	S	S	S	S	S	S	R	-
PaC20	non-MDR	S	S	S	S	S	S	S	S	S	S	S	S	R	-
PaC21	XDR	S	R	R	R	R	R	R	R	R	R	R	S	R	Neg
PaC23	non-MDR	S	S	S	S	S	S	S	S	S	S	S	S	S	-
PaC25a	non-MDR	S	S	S	S	S	S	S	S	S	S	S	S	R	-
PaC25b	non-MDR	S	S	S	S	S	S	S	S	S	S	S	S	R	-
PaC26	XDR	R	R	R	R	R	R	R	R	R	R	R	S	R	Neg
PaC27	non-MDR	S	S	S	R	S	S	S	S	S	S	S	S	R	Neg
PaC28	MDR	S	S	S	S	S	S	S	R	S	R	S	S	R	Neg
PaC29	non-MDR	S	S	S	S	S	S	S	S	S	S	S	S	R	-
PaC30	non-MDR	S	S	S	S	S	S	S	S	S	S	S	R	S	-
PaC31	MDR	S	S	S	R	R	S	S	R	R	R	S	S	R	Neg
PaC33	XDR	R	R	R	R	R	R	R	R	R	R	R	S	R	Neg
PaC34	MDR	R	R	R	R	R	R	R	R	R	S	R	S	S	Pos
PaC35	MDR	I	R	R	R	R	R	R	R	R	S	R	S	S	Pos
PaC36a	XDR	S	R	R	R	R	R	R	R	R	R	R	S	R	Neg
PaC36b	MDR	S	R	R	R	R	S	S	R	R	S	R	S	R	Neg
PaC37	XDR	S	R	S	R	R	R	R	R	R	R	R	S	R	Neg
PaC38	XDR	S	R	R	R	R	R	R	R	R	R	R	S	R	Pos
PaC39	non-MDR	S	S	S	S	S	S	S	S	S	S	S	S	R	-
PaC41	non-MDR	S	S	S	S	S	S	S	S	S	S	S	S	S	-
PaC42	MDR	S	S	S	R	R	R	R	S	S	R	R	S	R	Neg
PaC56	MDR	S	S	S	R	R	R	R	S	S	R	S	S	R	Neg
PaC43	XDR	S	R	R	R	R	R	R	R	R	S	R	S	R	Pos
PaC44	non-MDR	S	I	S	S	S	S	S	S	S	S	S	S	R	-
PaC45	XDR	S	R	R	R	R	R	R	R	R	R	R	S	R	Neg
PaC46	MDR	S	S	S	R	R	I	S	S	R	S	S	S	S	Neg
PaC47	XDR	S	R	R	R	R	R	R	R	R	S	R	S	R	Neg
PaC48	non-MDR	S	S	S	S	S	S	S	S	S	S	S	S	R	-
PaC54	non-MDR	S	S	S	S	S	S	S	S	S	S	S	S	R	-
PaC50	non-MDR	S	S	S	S	S	S	S	S	S	S	S	S	R	Neg
PaC53	non-MDR	S	S	S	S	S	S	S	S	S	S	S	S	R	-
PaC55	non-MDR	S	S	S	R	R	I	S	S	S	S	S	S	S	Neg
PaC57	non-MDR	S	S	S	S	S	S	S	S	S	S	S	S	R	-

^a^The isolates were classified according to the resistance pattern, as multidrug-resistant (MDR, non-susceptible to ≥1 agent in ≥3 antibiotic categories), extensively drug-resistant (XDR, non-susceptible to ≥1 agent in all but ≤2 antibiotic categories) and non-multidrug resistant (non-MDR).

^b^Antibiotics tested: Amikacin (AMK), Gentamicin (GEN), Tobramycin (TOB), Imipenem (IMP), Meropenem (MEM), Ceftazidime (CAZ), Cefepime (FEP), Ciprofloxacin (CIP), Levofloxacin (LVX), Aztreonam (ATM), Piperacilin/Tazobactam (TZP), Colistin (COL), Fosfomycin (FOS), Imipenem-EDTA (IMP-EDTA). Results are indicated as *S* sensitive, *I* intermediate, *R* non-susceptibility, *Neg* negative, *Pos* positive, *-* not applicable.

### MLST analysis

A total of 2,882 nucleotides were analysed for the 56 isolates: 390 bp for *acsA*, 498 for *aroE*, 373 bp for *guaA*, 442 bp for *mutL*, 366 bp for *nuoD*, 370 bp for *ppsA* and 443 bp for *trpE*. The number of polymorphic sites in the seven loci studied varied from 69 (*aroE*) to 11 (*nuoD*) in the 56 isolates studied. The number of alleles oscillated from 20 for *acsA* to 6 for *nuoD*. The *gua*A and *trpE* genes exhibited 15 different alleles, *mutL* and *pps*A exhibited 14 different alleles and *aroE* exhibited 10 different alleles. The allelic and nucleotide diversity are shown in Table 3. The *acsA*, *aroE*, *mutL* and *ppsA* genes displayed new alleles not previously described. The three isolates with two different colony morphologies presented the same allelic profile, although one of them (PaC36a and PaC36b) had different antibiotic susceptibility profiles. The allelic profile for the different isolates and for each gene analysed are given in Table 1. The new alleles and the new sequence types not previously described are indicated with an asterisk mark. The MLST analysis of the 56 isolates showed 32 different sequence types.

**Table 3:** **Genetic diversity of the selected loci among the *Pseudomonas aeruginosa *isolates analysed in this study**

**No of isolates**	**Locus**	**Fragment length (bp)**	**No. of alleles**	**Gene diversity ± SD**	**No. of polymorphic sites**	**Avg. number of nucleotide differences**	**Nucleotide diversity ± SD**
56	*acsA*	390	20	0.904 ± 0.027	23	5.15325	0.01321 ± 0.00095
56	*aroE*	498	10	0.827 ± 0.025	69	9.10909	0.01829 ± 0.00507
56	*guaA*	373	15	0.868 ± 0.034	14	2.62013	0.00702 ± 0.00062
54	*mutL*	442	14	0.764 ± 0.055	28	3.16702	0.00717 ± 0.00169
56	*nuoD*	366	6	0.642 ± 0.048	11	1.52922	0.00418 ± 0.00081
56	*ppsA*	370	14	0.879 ± 0.024	39	4.61364	0.01247 ± 0.00347
56	*trpE*	443	15	0.876 ± 0.023	19	4.50260	0.01016 ± 0.00076

**Figure 1:**
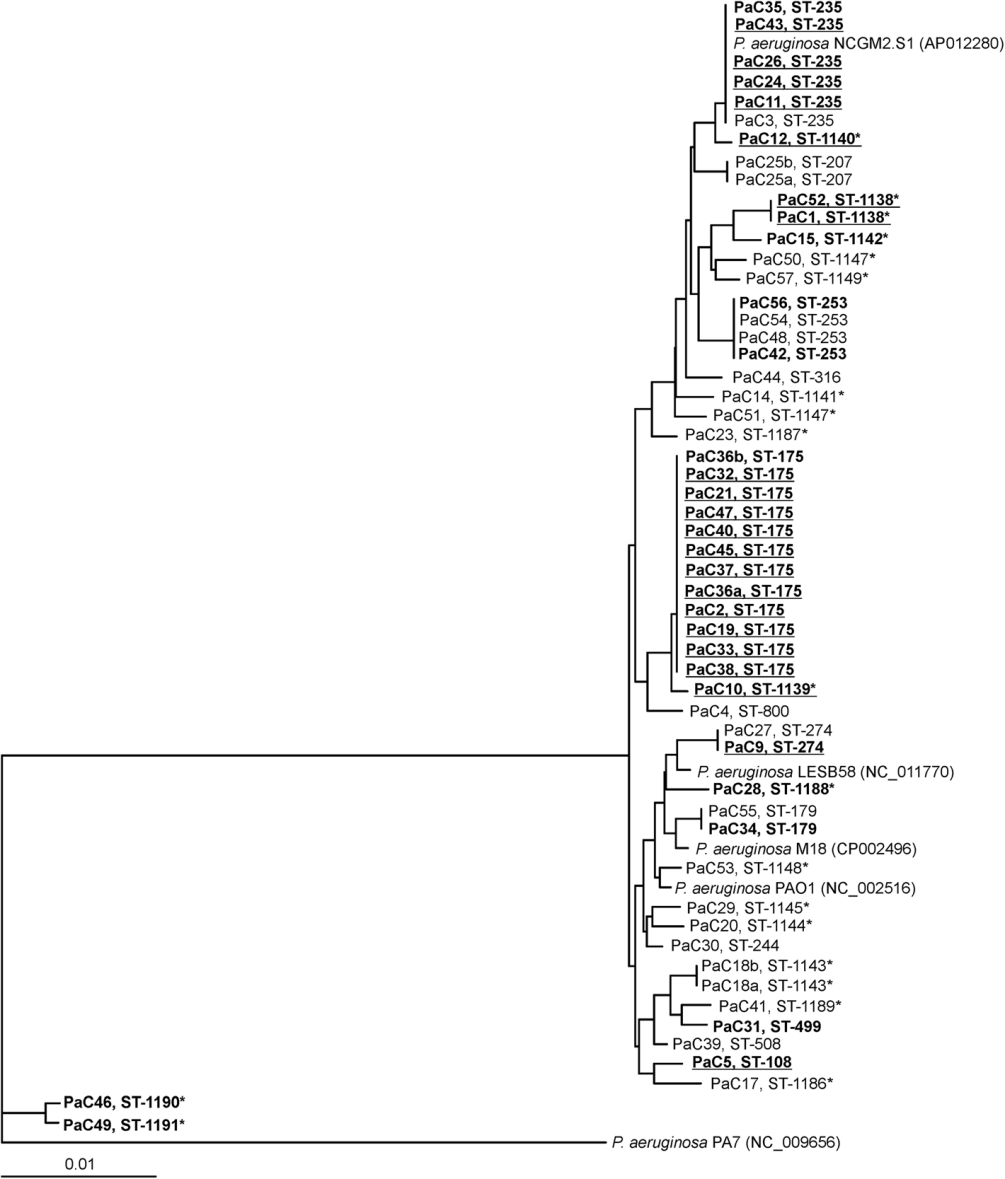
**Concatenated phylogenetic tree showing the molecular evolutionary relationships of the seven genes analysed (*acsA*, *aroE*, *guaA*, *mutL*, *nuoD*, *ppsA *and *trpE*) between the studied clinical *Pseudomonas aeruginosa *isolates.** The antibiotic profile is indicated in the figure: the MDR isolates are labelled in bold and the XDR isolates are indicated in bold and underlined. Clinical strains PaC7 and PaC16 are not included in the phylogenetic tree. Asterisk mark (*) indicates the new sequence types described in this study.

**Figure 2:**
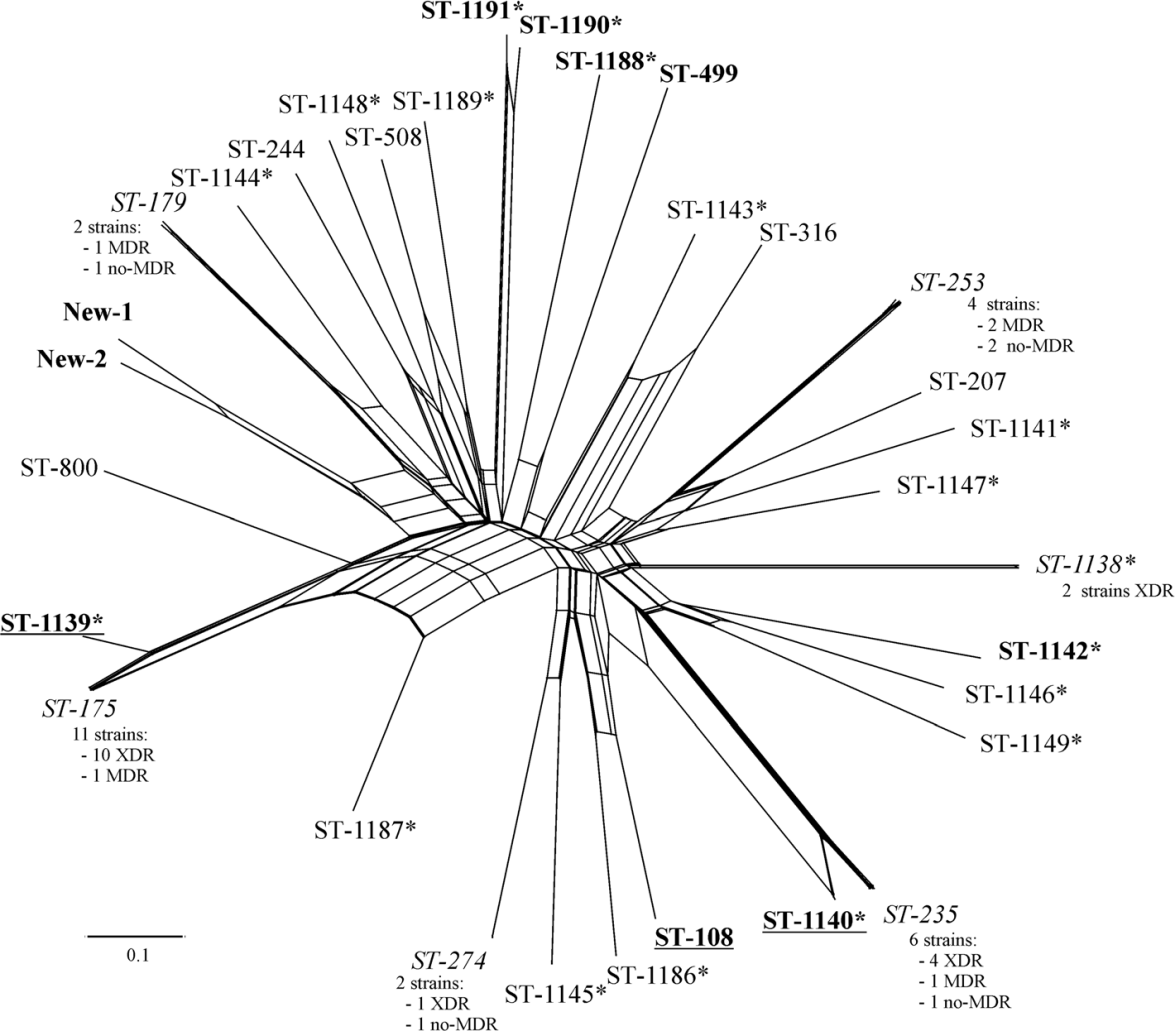
**SplitsTree showing the distribution of all of the sequence types obtained for the clinical *Pseudomonas aeruginosa *isolates studied.** The SplitsTree was based on the analysis of the allelic profiles of the *acsA*, *aroE*, *guaA*, *mutL*, *nuoD*, *ppsA* and *trpE* genes. The MDR isolates are labelled in bold and the XDR isolates are indicated in bold and underlined. The sequence types represented by more than one isolate are indicated in italic font. Asterisk mark (*) indicates the new sequence types described in this study.

Individual phylogenetic trees for each gene were constructed and, to build a more robust phylogeny, a concatenated analysis considering the seven genes was also performed (Figure 1). Two isolates with mucoid phenotype, PaC7 and PaC16, both isolated from the same patient (number 6), were not included in the analysis because we were unable to amplify and sequence the *mutL* gene. All of the clinical isolates studied, except PaC46 and PaC49, were related with a similarity between 98.5 - 100%. PaC46 and PaC49, belonged to the same clonal complex and shared a 99.8% similarity between them, less than 95.8% with the other clinical isolates and 95.7% with *P*. *aeruginosa* PA7, considered to be an outlier of the species [15]. The corresponding genes of *P*. *aeruginosa* PA7 and PAO1 have a similarity of 91.6%, and this percentage is lower when other species of the genus were considered. A SplitsTree was constructed with all of the isolates analysed (Figure 2), and recombination was observed. The most abundant sequence types observed were ST-175, ST-235 and ST-253.

### Patients and antibiotic resistance pattern

Thirty-five isolates were single isolates (one per patient), and, in seven patients, more than one isolate of *P*. *aeruginosa* was obtained during the two-month period studied (patients 1 and 8, four isolates each; patients 6, 9, 29, 32 and 38, two isolates each) (see Table 1). In two patients (9 and 38), all of the isolates studied belonged to the same ST and had the same antibiotic resistance profile. Isolates with different STs were isolated from three patients (patients 1, 6 and 8). Four isolates were isolated from patient 1 during the time of the study: PaC1 and PaC52 from a wound sample, and PaC49 and PaC51 from rectal smear. PaC1 and PaC52, were isolated with one month of difference, and belonged to the same ST and showed the same antibiotic resistance profile with the exception of gentamicin (intermediate susceptibility). PaC49 and PaC51 were assigned to different STs and showed differences in the antibiotic resistance profile. Patient 6 showed the same antibiotic profile (with the exception of meropenem). Four isolates with slight differences in the antibiotic profile were recovered from patient 8 (PaC10 and PaC19 from urine samples were isolated with three days of difference, PaC32 from a rectal smear and PaC40 was of respiratory origin). Isolate PaC10 was assigned to a different ST based on differences in *guaA* allele, although it belonged to the same clonal complex. Two isolates were isolated the same day from patient 29 from two different samples (catheter and blood); both of the isolates showed the same ST but presented differences in their antibiotic profile and in the production of MBLs, as detected by phenotypic methods. Two isolates of patient 32 obtained from different origins with two weeks of difference showed differences in piperacilin/tazobactam-susceptibility, but belonged to the same ST (see Table 1 and 2).

### Population structure and susceptibility to antibiotics

From the 56 isolates analysed, 23 were non-MDR and 33 were multiresistant (MDR or XDR). The non-MDR isolates were singleton STs, with the exception of ST-235 and ST-253. From the 56 isolates, 32 isolates were carbapenem-non-susceptible (57.1%) and 15.6% of them were MBL-positive. From those isolates, one was non-susceptible to only imipenem, and thirty-one were non-susceptible to both (isolate PaC16 showed intermediate resistance to meropenem). The 32 carbapenem-non-susceptible isolates were distributed into 15 sequence types: ST-175 (12 isolates), ST-235 (3), ST-179 (2), ST-253 (2), ST-274 (2), ST-108 (1), and ST-499 (1), and eight new sequence types (seven singletons and one with two isolates). Only four of these types (ST-175, ST-235, ST-253 and ST-274) were also described previously in the study of 16 Spanish hospitals [16].

No relations statistically significant could be established in our study between antibiotic resistance and other variables as sex, age of patients, sample origin or STs, probably because the low sampling potential. However, a statistically significant association was observed between the prevalent ST (ST-175) and multiresistant isolates (p = 0.003).

### Diversity analysis

To assess the extent of the diversity analysed in the study, a rarefaction curve was constructed. Despite the high diversity of the sequence types, the number of different sequence types referred to the number of isolates analysed did not reach a saturation curve, indicating that the diversity was higher than detected, a finding that was confirmed when the coverage index (C) was calculated (51%). Additional isolates should be analysed to ascertain the population structure of clinical *P*. *aeruginosa* present in our hospital completely. Diversity was evaluated using the Dominance (D), Shannon (H), Simpson and Evenness indices, and the values obtained for each index (0.075, 3.087, 0.925 and 0.684, respectively) indicate a highly diverse sample. However, when only the diversity of the multiresistant isolates (MDR and XDR) were considered, a softer saturation curve was detected and the coverage index was higher (62.5%), indicating that the diversity was better screened. This result was also supported by the diversity indices (D of 0.1621, H of 2.303, Simpson of 0.8379 and Evenness of 0.6255).

## Discussion

The role of *P*. *aeruginosa* as a pathogen and its implication in nosocomial outbreaks has been widely studied. The present study was focused on the analysis of the population structure and diversity of *P*. *aeruginosa* clinical isolates randomly chosen from their different patterns of antibiotic resistance in a single hospital. The isolates include different antibiotic non-susceptibility profiles (21.4% MDR, 37.5% XDR and 41.1% non-MDR).

The MLST analysis showed a high diversity, as reported in other previous studies. The 56 isolates were grouped into 32 different sequence types, 12 sequence types that were previously described (including 34 isolates) and 20 new ones (including 22 isolates). The singleton sequence types (26 isolates) corresponded mainly to the non-MDR isolates (16 isolates). Twenty-two of the isolates corresponded to new sequence types (not previously defined) of which 12 isolates were non-MDR, 6 isolates were MDR and 4 isolates were XDR. The clinical isolates studied showed a variable number of polymorphic sites and alleles, indicating the variability of the isolates selected. It is remarkable that we found the presence of new alleles (not previously described) of four genes, *acsA*, *aroE*, *mutL* and *ppsA*.

The analysis of the seven loci demonstrated that the prevalent STs were ST-175, ST-235 and ST-253. ST-175 is widely distributed worldwide [17] and is the isolate most frequently isolated in this study, with twelve isolates obtained from eight patients. This ST is also the most prevalent in the studies of García-Castillo et al. and Cholley et al. [16,17]. ST-175 has been reported as a contaminant of the hospital environment, a coloniser of respiratory secretions in cystic fibrosis patients, and has been associated with the multiresistant isolates of *P*. *aeruginosa*. All of the isolates included in this group were multiresistant (eleven XDR isolates and one MDR) and were sensitive to colistin, 90% to amikacin, 37% to aztreonam and nearly 10% to ceftazidime and cefepime. All of the isolates were resistant to the other antibiotics tested, and only one of them was MBL positive.

ST-235 is the second most frequently isolated sequence type, with six isolates (from five patients); four isolates were XDR, one isolate was MDR, and another was non-MDR. This ST has been involved in the dissemination of the genes encoding MBLs and has been associated to multiple resistance mechanisms [18], although Cholley et al. described strains with the same ST and none of them was MBL-positive [17]. Three of our five isolates were non-MBLs producers. In a previous study performed in another Majorcan hospital, ST-235 has been described as a VIM-13 producing β-lactamase [19]. ST-179, previously described in Mallorca as VIM-2 producer, was also MBL-positive [19].

The third most abundant sequence type was ST-253, with four isolates. These isolates were isolated from two patients; two isolates were MDR, and two were non-MDR.

Only one isolate was colistin-resistant, corresponding to ST-244 reported previously in Korea as the isolate most frequently colistin non-susceptible and sensitive to other antibiotics [20]. Our isolate was isolated in a mixed culture with *Morganella morganii* and *Serratia marcescens*, both inherently resistant to colistin.

The high discriminatory power of the MLST profiling allowed the differentiation among isolates obtained from the same patient at different dates and sampling sites. When the specimen was associated with the site of infection, the sequence type or clonal complex obtained and the antibiotic resistance profiles were the same.

## Conclusions

The present results indicate that *P*. *aeruginosa* isolates revealed a significant frequency of recombination and a panmictic net-like population structure, as was suggested by Kiewitz and Tümler [21]. The population structure of clinical *P*. *aeruginosa* present in our hospital indicates the coexistence of nonresistant and resistant isolates with the same sequence type. The multiresistant isolates studied are grouped in the prevalent sequence types found in other Spanish hospitals and at the international level, and the susceptible isolates correspond mainly to singleton sequence types.

## Competing interests

The authors declare no competing interests; financial or otherwise.

## Authors’ contributions

MG carried out the molecular genetic studies, participated in the sequence analysis and drafted the manuscript. MP carried out the molecular genetic analysis. MCG, VFB, and PDA carried out the isolation and phenotypic and the antibiogram analysis. AP performed the statistical analysis. MG, MCG, EGV and JL conceived the study. All co-authors participated in the design of the study and coordination and helped to the draft manuscript. All authors read and approved the final manuscript.
